# Effective generation mechanisms of tropical instability waves as represented by high-resolution coupled atmosphere–ocean prediction experiments

**DOI:** 10.1038/s41598-023-41159-5

**Published:** 2023-09-07

**Authors:** Takahiro Toyoda, L. Shogo Urakawa, Hidenori Aiki, Hideyuki Nakano, Eiki Shindo, Hiromasa Yoshimura, Yuma Kawakami, Kei Sakamoto, Akio Yamagami, Yusuke Ushijima, Yayoi Harada, Chiaki Kobayashi, Hiroyuki Tomita, Tomoki Tozuka, Goro Yamanaka

**Affiliations:** 1 Meteorological Research Institute, Japan Meteorological Agency, Tsukuba, Japan; 2 Institute for Space–Earth Environmental Research, Nagoya University, Nagoya, Japan; 3 Japan Meteorological Business Support Center, Tsukuba, Japan; 4 Faculty of Environmental Earth Science and Graduate School of Environmental Science, Hokkaido University, Sapporo, Japan; 5 Department of Earth and Planetary Science, Graduate School of Science, The University of Tokyo, Tokyo, Japan

**Keywords:** Climate sciences, Ocean sciences

## Abstract

Cusp-shaped fluctuations of the sea surface temperature (SST) front in the tropical Pacific, now known as tropical instability waves (TIWs), were discovered by remote sensing in the 1970s. Their discovery was followed by both theoretical and analytical studies, which, along with in situ observations, identified several possible generation mechanisms. Although modeling studies have shown that TIWs strongly influence the heat budget, their influence on local variations of realistically initialized predictions is not yet understood. We here evaluate a series of medium-range (up to ~ 10 days) coupled atmosphere–ocean predictions by a coupled model with different horizontal resolutions. Observational SST, surface wind stress, heat flux, and pressure data showed that representation of temporally and spatially local variations was improved by resolving fine-scale SST variations around the initialized coarse-scale SST front fluctuations of TIWs. Our study thus demonstrates the advantage of using high-resolution coupled models for medium-range predictions. In addition, analysis of TIW energetics showed two dominant sources of energy to anticyclonic eddies: barotropic instability between equatorial zonal currents and baroclinic instability due to intense density fronts. In turn, the eddy circulation strengthened both instabilities in the resolved simulations. This revealed feedback process refines our understanding of the generation mechanisms of TIWs.

## Introduction

Atmosphere and ocean general circulation models (GCMs) have been developed for understanding and predicting atmospheric and oceanic phenomena, and advances in their parameterizations and increased computational resources have contributed to improved predictions of weather and climate. Although operational weather predictions for periods of up to about 10 days (hereafter “medium range”^1^) have mainly been provided only by atmospheric models, recent studies have reported that coupled atmosphere–ocean models, which have previously been used mainly for predictions on seasonal and longer time scales, have advantages for representing medium-range weather events such as typhoon development^2, 3^. Advances in parameterizations, resolution, and initialization^4–8^ suggest that medium-range predictions can potentially be improved by using coupled models. However, further investigation of effective model configurations and initialization approaches is needed before coupled prediction systems can be used operationally.

To assess the performance of medium-range predictions by coupled models and their potential for future operational use, the Meteorological Research Institute (MRI) has conducted a series of coupled prediction experiments. To evaluate the impacts of horizontal resolution, two sets of grid coordinates have been prepared for each atmosphere and ocean component. The resulting predictions have been used to analyze processes of oceanic response and feedback to sequential typhoon passages^9^, and their prediction skills for basic atmospheric variables have been evaluated (Shindo et al. in preparation). Notably, prediction skills in the tropics have been significantly improved by increasing the resolution of the oceanic component; this improvement has been plausibly attributed to the representation of sea surface temperatures (SSTs) in relation to tropical instability waves (TIWs)^10–13^.

In the eastern tropical Pacific, TIWs are perturbations of the SST front with a typical period of 20–40 days and a wavelength of 1000–2000 km that propagate westward with a phase speed of approximately 0.5 m s^−1^ and are normally perceptible during August–December (except in El Niño years)^14–16^. TIWs play important roles in biogeochemical cycles^17–19^, and several TIW generation mechanisms have been proposed, including barotropic instability of the meridional shear between the South Equatorial Current (SEC) and the North Equatorial Countercurrent (NECC)^20–25^, between the SEC and the Equatorial Undercurrent (EUC)^22, 23, 26, 27^, or within the SEC^28, 29^, and baroclinic instability due to the density front north of the equatorial cold tongue^22, 23, 27–29^. These divergent mechanisms might be attributable partly to the existence of several types of disturbances^23, 29–31^ and partly to different representations of both the meridional shear of the zonal currents and the density gradient, depending on the model^32–34^. In terms of the (former) disturbance type, we focus here on cusp-shaped variations of the SST front (North Equatorial Front; NEF), which were originally identified as TIWs by satellite infrared observations^14^. In fact, the heat budget and, hence, SSTs in the NEF region are greatly affected by the TIW structure, whose representation depends on the resolution of the ocean model^10–13, 22^. In addition, it has been suggested that anticyclonic vortices (tropical instability vortices; TIVs) play an important role in generating the TIWs^24, 30, 33^. Thus, to properly represent the TIWs, it is necessary to resolve mesoscale TIVs.

Coupled models have already been used to investigate atmospheric responses (and feedbacks) to TIW-related SST perturbations^12, 13, 34, 35^. However, the impacts of TIWs on prediction fields starting from realistic initial conditions, including local variations, have not yet been examined. For example, prediction fields should be compared with observed day-to-day changes at a location where the SST front is passing. Such evaluation should advance our understanding of the applicability of coupled models to medium-range predictions.

Here, based on medium-range predictions, we investigate the reproduction of local variations in the timing and amplitude of the rapid changes associated with TIWs. In particular, the effects of the resolutions of both atmospheric and oceanic models are evaluated. Although, as described above, previous studies have indicated that mean (lower) SST biases are reduced by resolving the meridional heat transport associated with TIWs, whether local SST variations can be realistically reproduced in medium-range predictions is unclear. If realistic local variations are confirmed for the prediction fields, then the prediction series provides us a great opportunity for investigating active mechanisms of TIW generation based on the full-model variables and the realistic reproduction of actual TIWs. We thus discuss the processes leading to realistic local variations of TIWs by using a recently proposed energy flux diagnosis approach^36^. The energy analysis under the realistic conditions of the predictions provides useful information for understanding which mechanisms among the several mechanisms indicated in previous theoretical studies are dominant, and what model resolution is necessary to resolve them.

## Results

### Variations in surface variables associated with TIWs

The coupled atmosphere–ocean global prediction experiments consisted of 11-day integrations from initialized states at 00:00 UTC on every date in September 2018. This month was targeted because it had the most active TIWs in 2018, a non-El Niño year. Two resolutions of both the atmospheric component (about 55 km and 10 km) and the oceanic component (about 100 km and 10 km) were used, and a series of experiments was conducted by combining the relatively low and high resolution atmospheric components with the relatively low resolution (“LoLo” and “HiLo” experiments, respectively) or the relatively high resolution oceanic component (“LoHi” and “HiHi”, respectively). See “Methods” section for details of these experiments.

**Figure 1:**
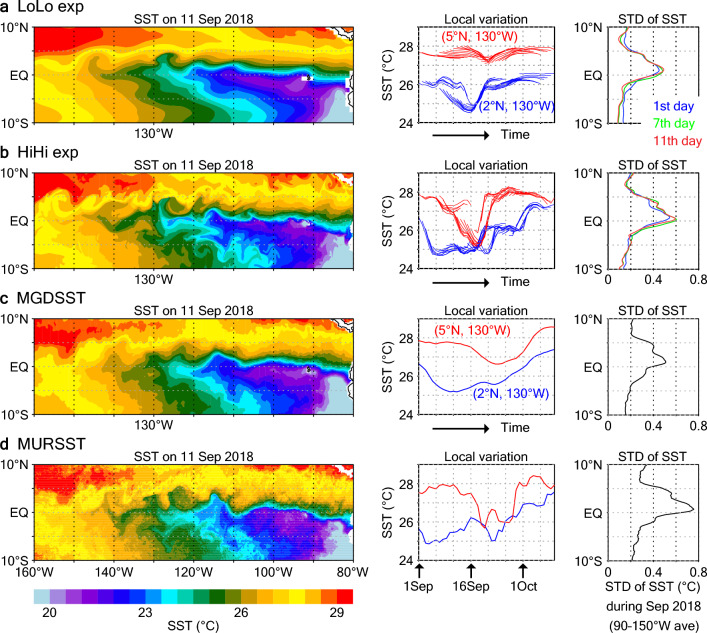
Predicted and observed SST distributions and variabilities. (Left) Horizontal SST distributions on 11 September 2018. (Middle) Time series of local SST variation at 2° N, 130° W (blue) and 5° N, 130° W (red). (Right) Meridional distributions of the daily STD of SST over 90–150° W during September 2018. (**a**) LoLo experiment. (**b**) HiHi experiment. (**c**) MGDSST. (**d**) MURSST. In (**a,b**), average SSTs on the 11th prediction day after initialization at 00:00 UTC 1 September 2018 (left), local variations of all predictions during the 11-day prediction period (middle), and monthly average STDs on the 1st, 7th, and 11th prediction days (blue, green, and red, respectively) (right) are plotted.

In both the LoLo and HiHi experiments, coarse-scale (wavelength ~ 1000 km) cusp-shaped SST distributions are represented north of the equator, as described in previous studies on TIWs; for example, northward cold-water intrusions are represented to the east of 130° W on 11 September 2018 (Fig. 1a,b; initialized at 00:00 UTC on 1 September). The consistency of these coarse-scale features with assimilated MGDSST data^37^ (Fig. 1c; operational observation product) suggests that in both experiments, the coarse-scale TIW structure is well constrained during the 11-day prediction period by the ocean initialization. In addition, the finer scale (wavelength of a few hundred kilometers; hence, mesoscale) variations with more intense SST gradients represented in the high-resolution observation product MURSST^38^ (Fig. 1d) are seen in HiHi. Amplitudes of the local SST variations (e.g., at 2° N, 130° W and 5° N, 130° W, points through which the represented TIW passes) differ greatly between LoLo and HiHi, and the latter variations are generally consistent with those of MURSST. Because the fine-scale variations are rather smoothed out in the MGDSST data as indicated by a previous study^39^ (e.g., Fig. 1c), these local variations are generated by the high-resolution ocean model dynamics. As a result, SST standard deviations (STDs) obtained for HiHi are larger by about 0.1 °C than those obtained for LoLo and MGDSST in the TIW region (about 0–6° N). Although the MURSST STDs are rather larger than the HiHi STDs, the MURSST product includes grid-scale (and day-to-day) noise, which can inflate STDs; in fact, the meridional STD distribution becomes more similar to that of HiHi if a constant of about 0.1 (as a background value) is subtracted over the domain (10° S–10° N). The SST gradient fields^40–42^ further underline the advantage of HiHi (Fig. S1). We do not think the fine-scale variations in HiHi are completely unconstrained, because they are affected by the coarse-scale TIWs reproduced locally by the initialization, as discussed below. Note that the large SST variability in the TIW region suggests that predictions of only atmospheric models forced by the persistent SST anomalies are insufficient for reproducing realistic atmospheric responses.

**Figure 2:**
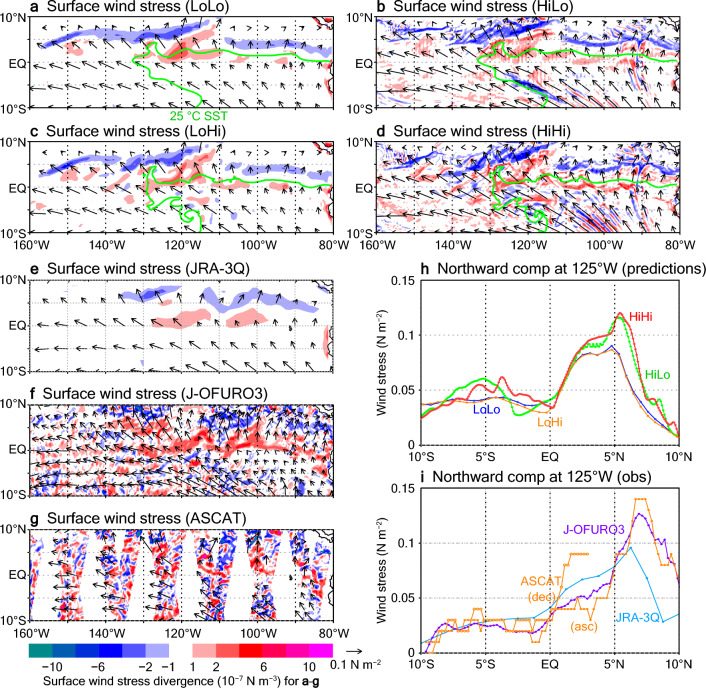
(**a–g**) Horizontal distributions of surface wind stresses (arrows) and their divergences/convergences (shading) on 11 September 2018. (**a**) LoLo experiment. (**b**) HiLo experiment. (**c**) LoHi experiment. (**d**) HiHi experiment. (**e**) JRA-3Q. (**f**) J-OFURO3. (**g**) ASCAT. In the predicted results (**a–d**), the 25 °C isotherm lines of SST (green lines) are also plotted. (**h**) Northward component of surface wind stress at 125° W for the prediction experiments. (**i**) Same as (**h**) but for the observational data. ASCAT data from both ascending (closed circles) and descending (open circles) passes are plotted.

Previous studies have investigated the structure of surface wind responses to TIW-induced SST variations and the effect of feedback of the surface winds to the ocean on the TIWs and also on El Niño–Southern Oscillation variability^43–49^. In our experiments, both the representation of the SST variations associated with TIWs and the atmospheric responses to those SST variations differ, depending on the resolution. On a broad scale, southeasterly winds covered the TIW region (Fig. 2, which shows conditions on 11 September 2018, the same date as the comparisons shown in Fig. 1). These winds passed over the NEF, from the colder side to the warmer side of the temperature front, and induced divergences of the wind stresses around the front, and convergences to the north, consistent with the findings of previous studies^43, 48^. This contrast was particularly strong around 120–130° W, where the NEF exhibited a southward fluctuation associated with the TIWs. Note that the filtered relationship between the SSTs and surface winds that extracts only the TIW-related variations, as presented in previous studies^35, 44, 46^, is not deducible from our relatively short term results. However, these broad-scale surface wind stress structures are basically the same in all four of our prediction experiments (Fig. 2a–d) and consistent with observational estimates (Fig. 2e–g). In addition, the predictions made with the relatively high resolution atmospheric model, HiLo and HiHi, showed sharp divergence and convergence structures (Fig. 2b,d). The peak values of the divergences/convergences, with magnitudes of about 5 × 10^−7^ N m^−3^, and of the wind stresses, with magnitudes above 0.1 N m^−2^, to the north of the NEF in these experiments (Fig. 2b,d,h) are consistent with the high-resolution retrievals of J-OFURO3^50^ and ASCAT^51^ (Fig. 2f,g,i). These results also support the advantage of using a high-resolution atmospheric model. On the other hand, surface wind stress differences due to the different ocean model resolutions are relatively small.

**Figure 3:**
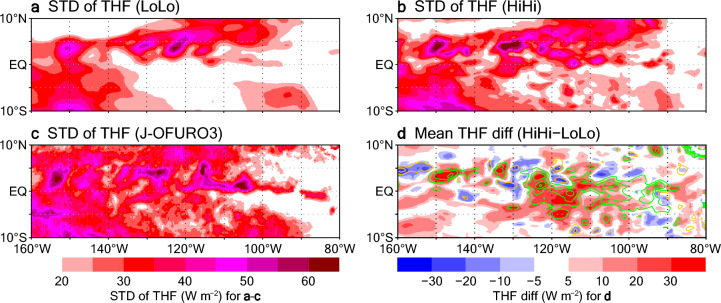
Horizontal distributions of daily THF STDs during September 2018. (**a**) LoLo experiment. (**b**) HiHi experiment. (**c**) J-OFURO3. (**d**) THF differences (HiHi minus LoLo; shading) averaged over September 2018, with mean SST differences (HiHi minus LoLo; contours) superimposed. Green (purple) contours denote positive (negative) SST differences; contour interval is 0.3 °C. In (**a,b,d**), averages on the 11th prediction day are plotted.

Variations in SST affect the atmosphere via surface fluxes. Here, we focus on the turbulent heat flux (THF; sensible plus latent heat fluxes). THF variability (as represented by its STDs) is larger in HiHi than in LoLo in the region with larger SST variability (Fig. 3a,b). In that region, the THF STD amplitudes in HiHi are generally similar to those in the observational surface flux product J-OFURO3^50^ (Fig. 3c). Around the TIW region, positive (upward) THF differences generally correspond to positive SST differences, and mean THFs differ between LoLo and HiHi by up to 30 W m^−2^ (Fig. 3d), which is non-negligible compared with the mean values of 40–140 W m^−2^. These THF differences are mostly explained by latent heat flux differences between LoLo and HiHi (not shown). Thus, the nonlinear dependency of the latent heat flux (via humidity) on SST, which is accompanied by large STDs, also contributes to the larger mean THF values in HiHi than in LoLo.

**Figure 4:**
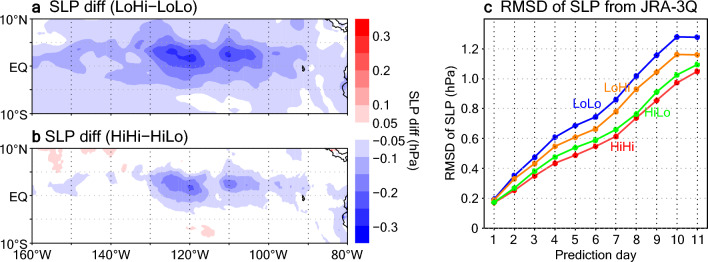
SLP differences in the predictions and the observational product. (**a,b**) SLP differences on the 11th prediction day averaged over September 2018. (**a**) LoHi minus LoLo. (**b**) HiHi minus HiLo. (**c**) RMSD of daily SLP relative to JRA-3Q in the active TIW region (0–6° N, 100–130° W) for each experiment. All predictions starting in September 2018 are used in (**c**).

Around the region with higher mean THFs, generated sea level pressure (SLP) values are lower in LoHi than in LoLo, and also lower in HiHi than in HiLo (Fig. 4a,b). The different distributions of SLP anomalies are due to different responses to surface forcing (THF) anomalies, which depend on the resolution of the atmosphere model^35^. Nevertheless, in both LoHi and HiHi, the lower pressure differences improve representation of the local SLP variations in the TIW region: root mean square differences (RMSDs) in SLP with respect to JRA-3Q reanalysis data^52^ are smaller in LoHi and HiHi than in LoLo and HiLo, respectively, during the prediction period (Fig. 4c). Therefore, the reproduction of fine-scale SST variations associated with TIWs by the high-resolution ocean model dynamics leads to lower SLPs through enhanced THFs and results in more realistic local SLP variations in medium-range predictions.

It should be noted that, although feedbacks to the oceanic fields of the above atmospheric responses exist (e.g., they appear as oceanic differences between LoHi and HiHi), the surface and subsurface temperature differences between the LoHi and HiHi experiments are small (magnitudes less than 0.2 °C) and not systematic in the TIW region during the experimental period, and they cannot be validated with the observational data. Thus, the feedbacks to the ocean take place on a longer time scale than the time scale on which the oceanic differences affect the atmosphere, as shown in this section. On the other hand, feedbacks of the TIW representations to the mean oceanic current field as well as to the temperature field, as reported in recent studies^53, 54^, may be represented at least partly in our experiments because the initialization experiments were conducted individually with the low- and high-resolution ocean models. In fact, the mean initialized oceanic fields exhibited marked differences in equatorial circulation, as well as in subsurface temperatures, that resulted from the resolution difference (Fig. S2). The weakening of the zonal currents in our results is consistent with these previous studies^53, 54^, although determination of whether the TIWs and other processes (e.g., the narrower equatorial upwelling in the high-resolution model) can be attributed to these differences is beyond the scope of this study.

Statistical evaluations of the lower atmospheric variables (SLP and the 850 hPa temperature) also showed that the prediction performance could be generally enhanced by the use of high-resolution atmosphere and ocean models (Figs. S3–S6). These validation results support that our experiments, in particular HiHi, well reproduce TIWs and associated mesoscale variations. Further validation of the upper atmospheric fields is beyond the scope of this study and will be discussed in another paper (Shindo et al., in preparation). Instead, we use this realistic simulation product to investigate TIW energetics. As described in the Introduction, the reproduction of local variations is highly important for identifying the actual effective TIW generation mechanisms among the many generation mechanisms that have been proposed based on simplified frameworks.

### Energetics of TIWs

Many previous analytical studies of TIWs have focused on equatorially trapped gravity waves, although planetary waves might also play a role in TIW generation, even in the tropics^22, 55–59^. A recently proposed analytical formulation that unifies the effects of both wave types is applicable to the diagnosis of the continuous three-dimensional energy flow structure from the equatorial region to higher latitudes, without meridional or vertical mode decomposition^36^. Here, the energy supply to the TIWs as represented in the previous section is diagnosed by using this formulation of the energy flux diagnostics (see “Methods” section).

**Figure 5:**
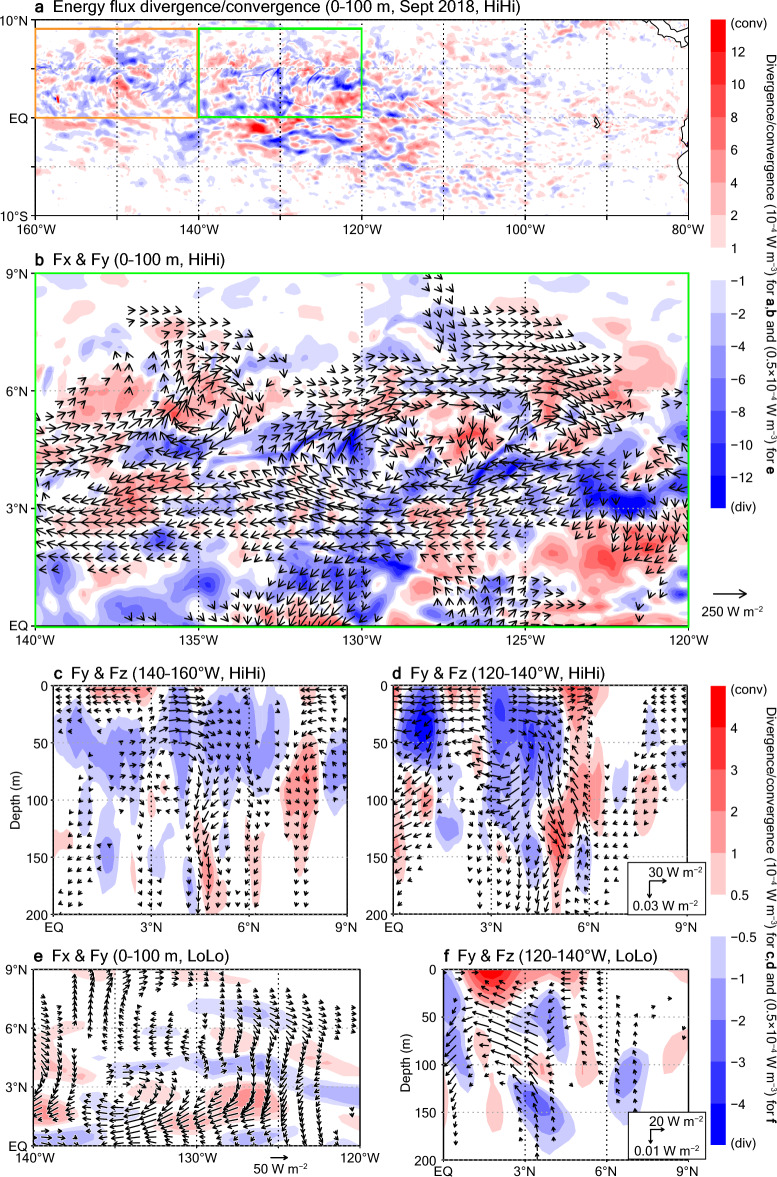
Eddy energy flux divergence and convergence distributions in HiHi and LoLo. (**a**) Horizontal distribution of divergences/convergences of three-dimensional energy fluxes averaged over 0–100 m depth. (**b**) Same as (**a**) but for the region indicated by the green box in (**a**). Horizontal components of the energy fluxes (arrows) are superimposed. (**c,d**) Zonal averages of the three-dimensional divergences/convergences (shading) and meridional and vertical components (arrows) of the energy fluxes in meridional cross sections. (**c**) 140–160° W (orange box in **a**). (**d**) 120–140° W (green box). In (**a–d**), results from HiHi averaged over September 2018 are shown. (**e,f**) Same as (**b,d**) but showing results from LoLo.

Monthly averaged surface-layer divergences and convergences of the energy fluxes indicate high eddy activity in the TIW region (Fig. 5a). Eddy energy supplies, corresponding to the divergences, are particularly large between 140 and 120° W (green box in Fig. 5a). Relatively large divergences are broadly distributed not only near the equator (about 0–2° N) but also between around 3 and 5° N (Fig. 5b). Energy streams with northward components stemming from the latter region converge at latitudes of about 5–7° N. The zonal mean field further underlines this northward energy flow structure (Fig. 5d), which extends from the surface down to the thermocline (about 100–150 m depth). It also shows that downward components are responsible for the deeper convergences; this result is consistent with previous studies indicating that in this region, the downward propagation of TIW-related waves affects conditions at these depths^27, 60, 61^.

Farther west (140–160° W; orange box in Fig. 5a), divergences and convergences are relatively small, although downward fluxes are still seen (Fig. 5c). Divergence bands are also seen in the low-resolution ocean model results, but the divergence amplitudes are smaller and the eddy energy radiates mainly southward, resulting in much smaller convergences to the north (Fig. 5e,f). Note that when the oceanic resolution is the same, the energy fluxes differ only slightly in our medium-range predictions (not shown); therefore, atmospheric feedbacks are not considered in this study. Thus, although the initialization can improve the representation of the cusp-shaped SST front variations at coarse scale in both low- and high-resolution ocean models, the energy supplies for the generation/intensification of the TIWs are greatly affected by fine-scale variations in the high-resolution ocean model.

**Figure 6:**
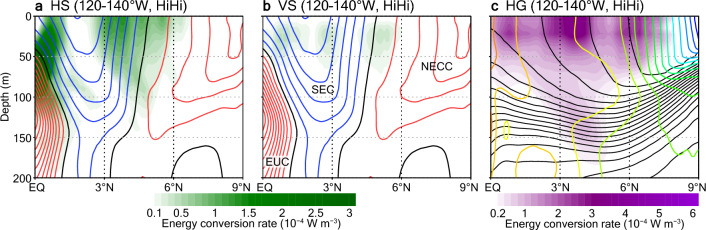
Mean energy conversion rate distributions in HiHi. (**a**) Rates of energy conversion from mean to eddy kinetic energy due to mean horizontal shear (HS; shading) and zonal velocities (contours, interval is 10 cm s^−1^; blue, black, and red indicate westward, zero, and eastward, respectively) averaged over 120–140° W during September 2018 from HiHi. (**b**) Same as (**a**) but for rates of energy conversion from mean to eddy kinetic energy due to mean vertical shear (VS). (**c**) Same as (**a**) but for rates of energy conversion from mean to eddy potential energy due to the mean horizontal density gradient (HG; shading) and potential temperature (black contours with 1 °C interval) and salinity (colored contours with 0.1 interval; cool (dark blue) to warm (red) colors indicate salinities from 33.7 to 35.2).

Eddy energy supplies in the divergence region are attributable to energy conversions from mean to eddy fields. Considering previous study results^23, 25–27, 35, 53, 54^, we investigate three energy sources (through barotropic and baroclinic instabilities): mean horizontal shear (HS), mean vertical shear (VS), and the mean horizontal density gradient (HG). The estimation of the energy conversion rates for these energy sources is described in “Methods” section. HS and HG make major contributions to energy supplies between 3 and 5° N, whereas the contribution of VS is minor there (Fig. 6). Large HS is confined to the regions between the EUC and SEC and between the SEC and NECC, as indicated previously^30, 33^. The energy flux analysis results indicate that the energy supplies in the northern (SEC–NECC) region effectively cause the SST front fluctuations (Fig. 5b,d).

**Figure 7:**
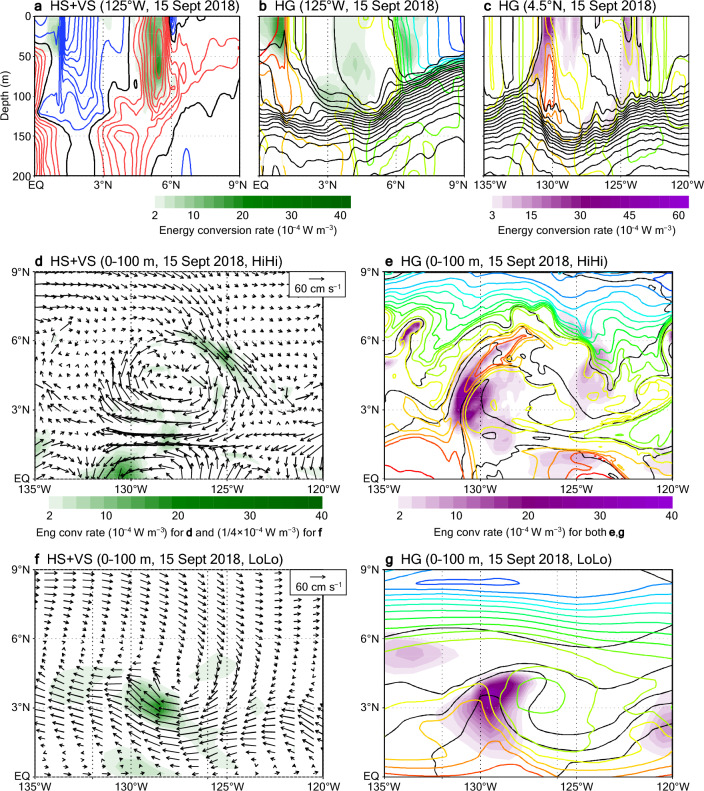
Snapshot energy conversion rate distributions in HiHi and LoLo. (**a**) Conversion rates of HS plus VS (shading) and zonal velocities (contours as in Fig. 6a) on a meridional cross section along 125° W on 15 September 2018. (**b**) Same as (**a**) but for conversion rates of HG (shading) and potential temperature and salinity (contours as in Fig. 6c). (**c**) Same as (**b**) but along 4.5° N. (**d**) Conversion rates of HS plus VS averaged over 0–100 m depth (shading) and horizontal velocities at 50 m depth (vectors) on 15 September 2018. (**e**) Conversion rates of HG averaged over 0–100 m depth (shading) and potential temperature and salinity at 50 m depth (contours as in **b**,**c**) on 15 September 2018. Results from HiHi are plotted in (**a–e**). (**f,g**) Same as (**d,e**) but showing results from LoLo.

A snapshot example highlights regions with instabilities associated with a mesoscale anticyclonic eddy (TIV centered at 4° N, 128° W on 15 September 2018; Fig. 7a–d). Shear instability (mainly due to HS) occurs on the southern and northern rims of the eddy, where relatively strong mean horizontal shears are associated with zonal currents (Fig. 7a,d). Baroclinic instability (due to HG) occurs on both the western and eastern sides of the eddy; relatively cold, salty equatorial water intrudes northward in the western part, whereas relatively warm, less-saline water intrudes southward in the eastern part, and both intrusions are accompanied by intense density fronts (Fig. 7b,c,e; see also Fig. S1).

Although the energy supplied by the HS and HG mechanisms in the southern and western parts, respectively, is similar between LoLo and HiHi, energy supplies in other parts are weak or absent in LoLo (Fig. 7f,g). In LoLo, the relatively weak northward intrusion in the western part does not approach the NECC region (> 5° N, approximately) but returns southwestward to the SEC region, consistent with the southward energy fluxes in this region (Fig. 5f).

**Figure 8:**
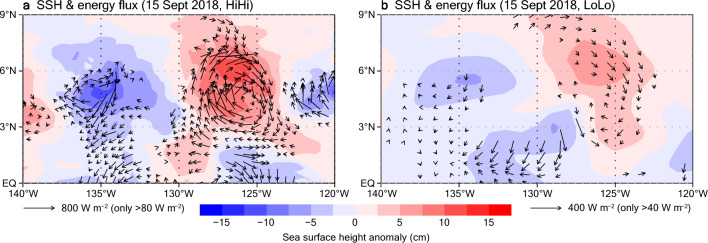
Horizontal distributions of SSH anomalies (shading) and horizontal energy fluxes (averaged over 0–100 m depth; arrows) on 15 September 2018. (**a**) HiHi. (**b**) LoLo.

As shown in Fig. 7d–g, more intense TIV circulation is represented in HiHi than in LoLo. Consistent with the findings of previous studies that detected TIVs from the satellite sea surface height (SSH) data^30, 33^, this difference is reflected in the SSH anomaly fields (Fig. 8); the SSH anomalies associated with the TIV are much larger in HiHi than in LoLo, and the maximum value in HiHi, which is > 10 cm, is consistent with the observed TIV values. In HiHi, energy fluxes with a northward component on the southwestern side of the TIV (around 3° N, 129° W) and those with a southward component on its north–northeastern side are directed toward the TIV that is characterized by high SSH anomalies, which can act to maintain the TIV (Fig. 8a). In contrast, LoLo represents much weaker energy fluxes toward the TIV on the northern side, and those on the southwestern side are opposite in direction (Fig. 8b). Hence, the fact that the TIV and associated energy fluxes are not well resolved in LoLo can explain the damped representation of mesoscale and local SST variations of the TIWs in LoLo (Fig. 1a) relative to those in HiHi (Fig. 1b). The process by which TIVs dominate eddy activity in this region and generate TIW variations is further discussed in the following section.

## Summary and discussion

In this study, we first evaluated a series of medium-range predictions by coupled atmosphere–ocean models with different horizontal resolutions in terms of variations related to TIWs. The results showed that, in addition to the large (about 1000 km) scale TIW variations as initialized (by data assimilation) in both low- and high-resolution ocean models, local (mesoscale) variations in observational SST, surface wind stress, surface heat flux, and surface pressure data are better represented by the high-resolution ocean model. These simulations with realistic local variations make it possible to investigate the energetics of the TIWs, in particular, the dominant mechanisms among several previously proposed mechanisms, which were mostly based on simplified frameworks or limited observations. Thus, we next analyzed the eddy energy fluxes and sources influencing the TIWs in the simulations. Our results indicate the importance of barotropic instability in the SEC and NECC region and of baroclinic instability in the NEF region in supplying eddy energy to the TIWs. More specifically, mesoscale eddies, that is, TIVs, are generated in the SEC–NECC region as a result of the barotropic and baroclinic instabilities, and the anticyclonic circulation of the TIVs induces northward intrusions of cold, saline water from the equatorial region on the western side, leading to the large-scale, cusp-shaped SST variations of the TIWs. Although our results might not be surprising, we consider that they add useful information to previously reported findings in determining which are the effective processes generating TIWs among those long discussed in the literature.

In our simulations with both low- and high-resolution ocean models, barotropic instability is seen on the northern rim of the SEC. A previous analytical study^25^, using a linear shallow water model, showed that a chain of anticyclonic and cyclonic eddies was the fastest growing mode that corresponded to the barotropic instability for the mean states in this region (similar to those in this study; Fig. S7). The anticyclonic warm-core TIV eddies to the north of the barotropic instability in our results are consistent with these previous study results, although the cyclonic eddies zonally aligned with the anticyclonic eddies are weaker in our results. It has been proposed^16^ that nonlinearity affects cyclonic and anticyclonic eddies differently: in anticyclonic eddies, because a centrifugal force acts in the same direction as the pressure force, an increased velocity is required for the Coriolis force to achieve balance; in addition, cyclonic eddies associated with an uplifted thermocline are more intensely affected by vertical mixing, which dissipates the eddies. Previous observational studies have already pointed out the importance of nonlinear effects (a large Rossby number) on TIVs^30, 33^, but the specific processes involved in TIV eddy energetics have not been described previously. In our high-resolution ocean model results, the baroclinic instability due to the density front (conversion of mean to eddy potential energy) seen on the western and eastern sides of the TIV is attributable to distortion of the density front by the eddy advection effect, and barotropic instability is seen on the northern side of the TIV, where the eastward NECC is intensified by the eddy circulation. In contrast, with the low-resolution ocean model, the density front intrusions are not well resolved; although baroclinic instability of a similar magnitude is seen on the southwestern side of the TIV, the barotropic instability on the northern side of the TIV is unclear, possibly because of weak TIV circulation. Supporting our high-resolution ocean model results, the importance of the mean to eddy potential energy conversion term in this region (3–6° N) was pointed out previously, based on in situ observation data^23^. Thus, the barotropic instability on the northern rim of the SEC (about 3–5° N) supplies the eddy energy for the TIVs to the north, and, in turn, the resolved eddy circulation of the TIVs strengthens the other instabilities. In other words, the distributions of the eddy energy sources and fluxes represented in the high-resolution ocean model results suggest that the above-described instabilities contribute to the TIV generation, whereas comparison of the low- and high-resolution ocean model results suggests that there is feedback of the TIV circulation to the instabilities. In addition, the finding that the cyclonic eddies are weaker in magnitude than the anticyclonic eddies can be attributed to weaker nonlinear effects. Note that previous observational studies^30, 62^ have also reported the existence of cyclonic eddies in this region. Thus, our results are generally consistent with previous theoretical and observational studies, and they also underline that the cusp-shaped TIWs are effectively induced by the anticyclonic TIVs having stronger circulation than the cyclonic eddies. This feedback process for TIV development, which is highlighted by our comparison of the eddy energy supply (mean to eddy energy conversion) and flux fields between the low- and high-resolution predictions, refines our understanding of the generation mechanisms of TIWs.

Unfortunately, our prediction experiments were limited to one month (September 2018). Thus, determination of whether our results are applicable more broadly remains for future work. However, it was possible to represent positive impacts on the local variations of surface variables by using the high-resolution ocean model, and further evaluation of the atmospheric responses to these resolved surface variations is ongoing (Shindo et al., in preparation). These results support that high-resolution coupled models have advantages for medium-range predictions, in line with other recent developments and studies^2, 3^. Moreover, they demonstrate the potential of energy source and flow analyses using realistic simulation fields for investigating TIW generation, in which multiple mechanisms interact in a rather complicated manner. Our approach enables us to represent for the first time an instance of the convergence of the eddy energy generated by these multiple mechanisms into a TIV eddy, leading to TIW disturbances. We are encouraged that by further long-term and detailed analyses it will be possible to clarify how the converged eddy energy contributes to TIV generation. In future work, we plan to investigate whether this TIV generation process due to eddy energy convergence can be quantitatively resolved by our high-resolution ocean model.

## Methods

### Coupled atmosphere–ocean prediction experiments

We conducted a series of coupled atmosphere–ocean prediction experiments with atmosphere and ocean GCMs developed in the MRI of the Japan Meteorological Agency (JMA)^9^. For the atmosphere model, both TL319 (resolution about 55 km) and DL1919 (resolution about 10 km with double Fourier series^63^) grid coordinates were used, and JMA’s operational analysis fields were used for the initial conditions. For the ocean model, both non-eddy-resolving and eddy-resolving grid coordinates were used: the resolution of the former is 1° zonally and 0.3–0.5° meridionally (finer around the equator; a tripolar grid coordinate system was adopted north of 64° N)^64^, whereas the resolution of the latter is 1/11° zonally and 1/10° meridionally. The oceanic initial conditions were provided for the simulations of each resolution by 3-dimensional variational data assimilation^65^. Hence, we used four sets of grid coordinates for the coupled model: The “LoLo” and “LoHi” experiments were conducted with the TL319 atmosphere model and the non-eddy-resolving or eddy-resolving ocean model, respectively, whereas the “HiLo” and “HiHi” experiments were conducted with the DL1919 atmosphere model and the low- or high-resolution ocean model, respectively. For each model, 11-day-long predictions with initial times of 00:00 UTC for all days during September 2018 were obtained. This experimental series comprises version 1.7 experiments of an on-going MRI/JMA project.

### Reference data

We used both daily operational SST data with a resolution of 1/4° from the JMA (MGDSST^37^, operational mode) and a high-resolution (resolution 1/100°) SST product (MURSST^38^); both products are based on satellite microwave observations.

We used daily satellite-based estimates of wind stresses and sensible and latent heat fluxes at the oceanic surface with a resolution of 1/4° (J-OFURO3 V1.1^50^). In this study, only standard deviations (STDs) of the turbulent heat fluxes (THFs) are compared with the J-OFURO3 product, because the mean values might be affected by the differences in the assumptions used for calculating the surface fluxes (with bulk formulae). In fact, an intercomparison study of observational surface flux products (including J-OFURO3) has indicated mainly high correlations between these products and in situ observations with the typical biases for the individual products^66^.

We used daily wind stress retrievals with a resolution of 1/4° based on ASCAT^51^ 25 km scatterometer swath observations provided by E. U. Copernicus Marine Service Information.

We used SLP analyses with a resolution of 1.25° from the most recent JMA atmospheric reanalysis dataset (JRA-3Q^52^), which is the successor to the JRA-55 reanalysis^67^.

### Energy flux diagnostics

The level-2 expression of the three-dimensional eddy energy flux ***F*** is as follows^36, 68^:

$${\varvec{F}}=\left[{F}_{x},{F}_{y},{F}_{z}\right]={\rho }_{0}\cdot \left[\overline{{u }^{\prime}\Phi }+\frac{\partial }{\partial y}\left(\frac{\overline{\Phi {\varphi }^{app}}}{2}\right),\overline{{v }^{\prime}\Phi }-\frac{\partial }{\partial x}\left(\frac{\overline{\Phi {\varphi }^{app}}}{2}\right),\overline{{w }^{\prime}\Phi }\right],$$

where $\left(u^{\prime},v^{\prime},w^{\prime}\right)$ are eddy velocities in the *x*-, *y*-, and *z*-directions, respectively; $\Phi$ is geopotential; and ${\varphi }^{app}$ is the pseudostreamfunction of a rotation flux derived from the inversion of potential vorticity $q=\frac{\partial {v}^{\prime}}{\partial x}-\frac{\partial {u}^{\prime}}{\partial y}+\frac{\partial }{\partial z}\left(\frac{f}{{N}^{2}}\frac{\partial\Phi }{\partial z}\right)$ as

$$\left(\frac{{\partial }^{2}}{{\partial x}^{2}}+\frac{{\partial }^{2}}{{\partial y}^{2}}\right){\varphi }^{app}+\frac{1}{{\rho }_{0}}\frac{\partial }{\partial z}\left[\frac{{\rho }_{0}}{{N}^{2}}\frac{\partial }{\partial z}\left({f}^{2}{\varphi }^{app}\right)\right]=q.$$

${\rho }_{0}$ is a reference density, $f$ is the Coriolis parameter, and $N$ is the buoyancy frequency. Eddy fields are defined as anomalies from mean fields.

Mean fields used in this study are the averages of the initialization experiment results over a 3-month period centered on the target month (i.e., August–October 2018; Fig. S7). The tropical Pacific is known to exhibit intense seasonal and interannual variabilities^69, 70^. Hence, we consider the above definition of mean states to be a reasonable compromise that includes several TIWs and suffers little from seasonal changes.

For the time evolution, taking into consideration the continuity of the time series, we use daily mean time series of the target month composed of the 1st to 5th prediction days starting at 00:00 UTC on 1, 6, 11, 16, 21, and 26 September 2018.

### Energy conversion from mean to eddy fields

We investigated three mean energy sources as eddy generation mechanisms: mean to eddy kinetic energy conversion due to mean horizontal shear, HS; mean to eddy kinetic energy conversion due to mean vertical shear, VS; and mean to eddy potential energy conversion due to a horizontal gradient of potential density, HG. The energy conversion rates due to these processes are expressed as

$$\mathrm{HS}=-\overline{{\mathbf{u} }^{\prime}{v}^{\prime}}\cdot \frac{\partial \overline{\mathbf{u}}}{\partial y }-\overline{{\mathbf{u} }^{\prime}{u}^{\prime}}\cdot \frac{\partial \overline{\mathbf{u}}}{\partial x },$$

$${\text{VS}}=-\overline{{\mathbf{u} }^{\prime}{w}^{\prime}}\cdot \frac{\partial \overline{\mathbf{u}}}{\partial z },$$

$$\mathrm{HG}=-\frac{{g}^{2}}{{\rho }_{0}^{2}{N}^{2}}\left(\overline{{u }^{\prime}{\rho }^{\prime}}\cdot \frac{\partial \overline{\rho }}{\partial x}+\overline{{v }^{\prime}{\rho }^{\prime}}\cdot \frac{\partial \overline{\rho }}{\partial y}\right),$$

where $\mathbf{u}^{\prime}$ and $\overline{\mathbf{u} }$ are eddy and mean horizontal velocity vectors, respectively; $g$ is gravitational acceleration; and $\rho^ {\prime}$ and $\overline{\rho }$ are eddy and mean potential density, respectively^71, 72^.

### Supplementary Information

Supplementary Figures.

## Data Availability

The MGDSST data are available at http://ds.data.jma.go.jp/gmd/goos/data/rrtdb/jma-pro/mgd_sst_glb_D.html. The MURSST data are available from the NASA website at https://podaac.jpl.nasa.gov/dataset/MUR-JPL-L4-GLOB-v4.1. The J-OFURO3 V1.1 dataset is available at https://doi.org/10.20783/DIAS.612. The ASCAT data are available at https://doi.org/10.48670/moi-00182. The JRA-3Q reanalysis is available at https://jra.kishou.go.jp/JRA-3Q/index_en.html. The prediction experiment results used in this study are available from the corresponding author upon reasonable request.
