# Supplementary material for: Effective generation mechanisms of tropical instability waves as represented by high-resolution coupled atmosphere–ocean prediction experiments
Source: Sci Rep. 2023 Sep 7;13:14742. doi: 10.1038/s41598-023-41159-5 (PMC10485077; doi:10.1038/s41598-023-41159-5)
Supplement: Supplementary file 1 — Supplementary Figures. [file 41598_2023_41159_MOESM1_ESM.pdf]

Supplementary information for

Effective generation mechanisms of tropical instability waves represented by high-resolution coupled atmosphere–ocean prediction experiments

Takahiro Toyoda, L. Shogo Urakawa, Hidenori Aiki, Hideyuki Nakano, Eiki Shindo, Hiromasa Yoshimura, Yuma Kawakami, Kei Sakamoto, Akio Yamagami, Yusuke Ushijima, Yayoi Harada, Chiaki Kobayashi, Hiroyuki Tomita, Tomoki Tozuka, Goro Yamanaka

Contents: Supplementary Figure S1–S7

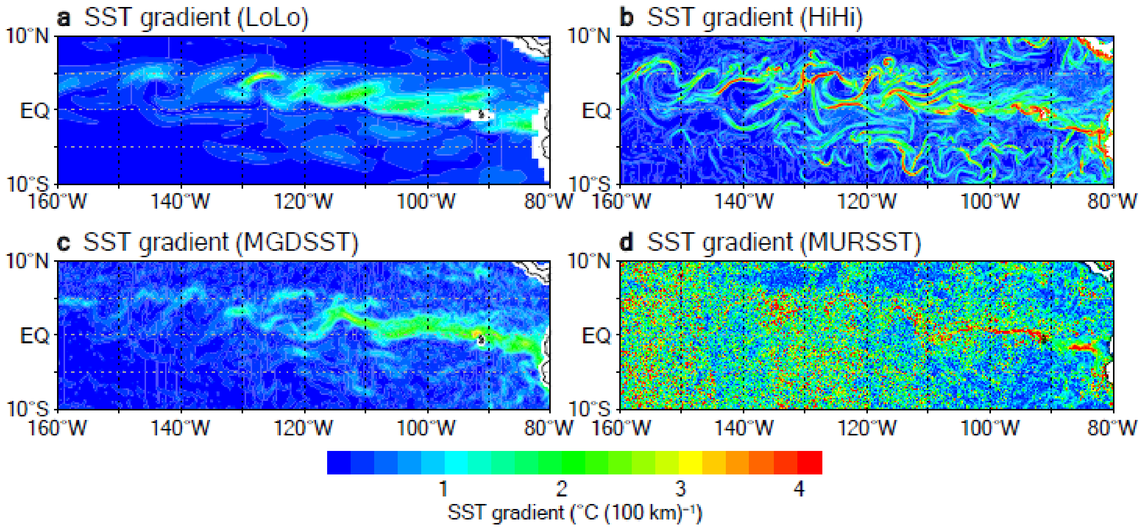

Figure S1. Distributions of SST gradients on 11 September 2018. (**a**) LoLo experiment. (**b**) HiHi experiment. (**c**) MGDSST. (**d**) MURSST. In addition to the generally meridional gradients represented as zonal bands in LoLo and MGDSST, the fine-scale structure in HiHi shows zonal gradients associated with the meridional intrusions discussed in the Energetics of TIWs section. Grid-scale noise, by inflating STDs, obscures the gradients in MURSST.

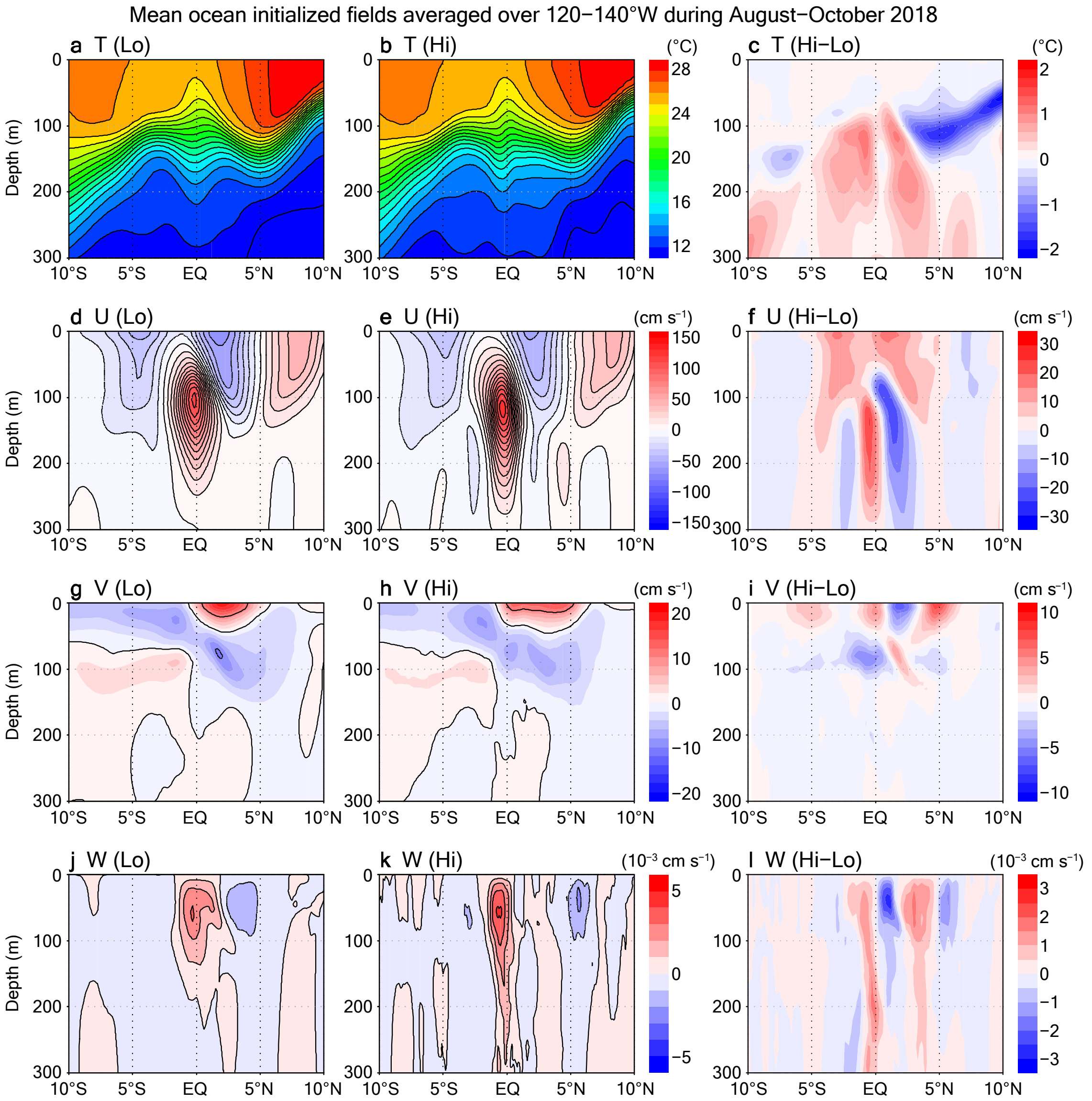

Figure S2. Mean ocean initialized fields obtained with the low-resolution (Lo; **a, d, g, j**) and high-resolution (Hi; **b, e, h, k**) ocean models averaged over 120–140°W during August–October 2018. Differences (Hi minus Lo) are also plotted (**c, f, i, l**). (**a–c**) Potential temperature. (**d–f**) Zonal velocity. (**g–i**) Meridional velocity. (**j–l**) Vertical velocity (positive upward).

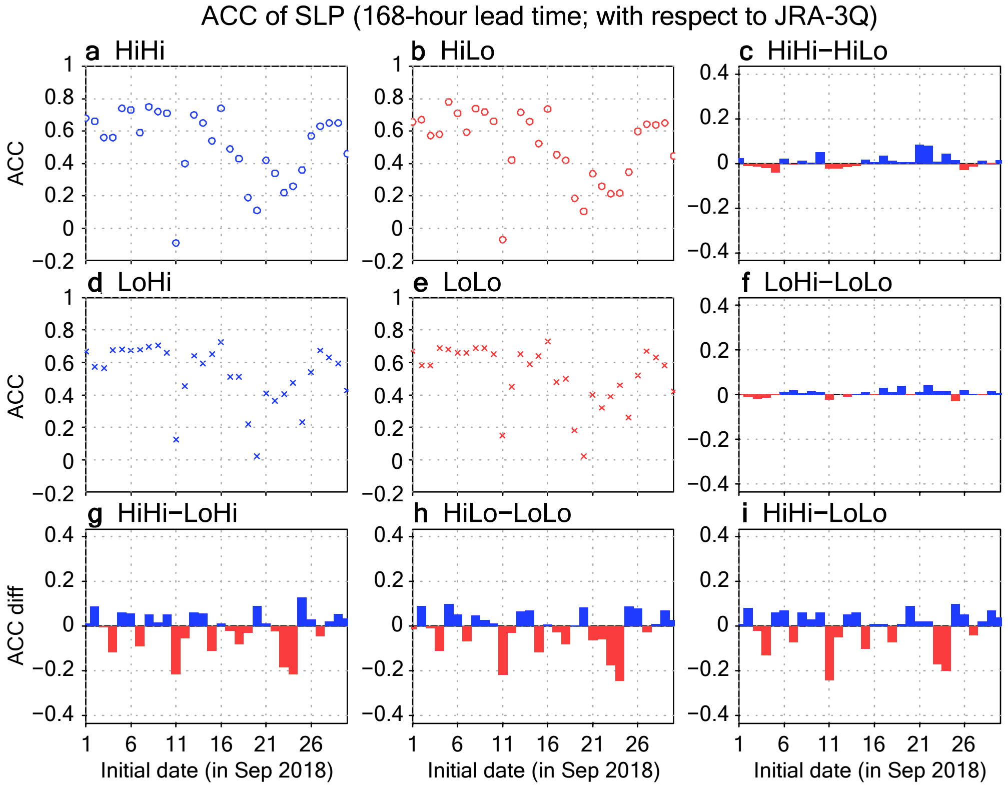

Figure S3. Time series of anomaly correlation coefficient (ACC) scores of sea level pressure (SLP) with respect to the JRA-3Q reanalysis for our predictions of 168-h lead time by the different resolution coupled models and their differences. (**a**) HiHi. (**b**) HiLo. (**c**) HiHi minus HiLo. (**d**) LoHi. (**e**) LoLo. (**f**) LoHi minus LoLo. (**g**) HiHi minus LoHi. (**h**) HiLo minus LoLo. (**i**) HiHi minus LoLo. These scores were calculated for the central–eastern tropical Pacific (20°S–20°N, 80–180°W).

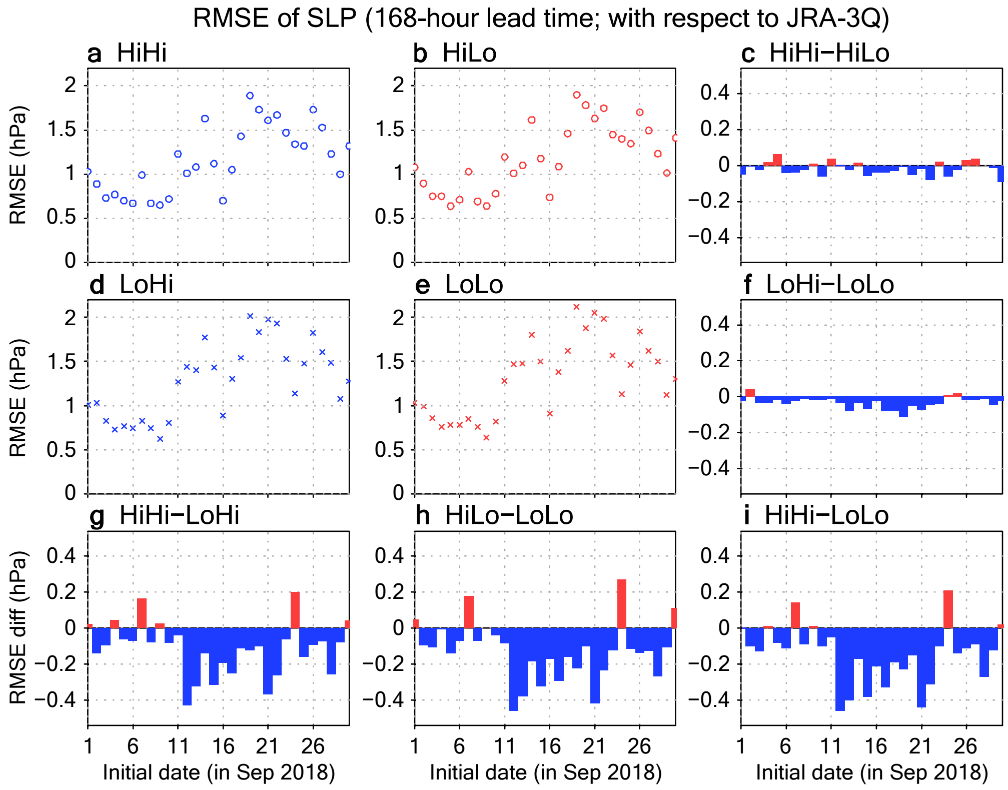

Figure S4. Same as Fig. S3 but for the root mean square error (RMSE) of sea level pressure (SLP).

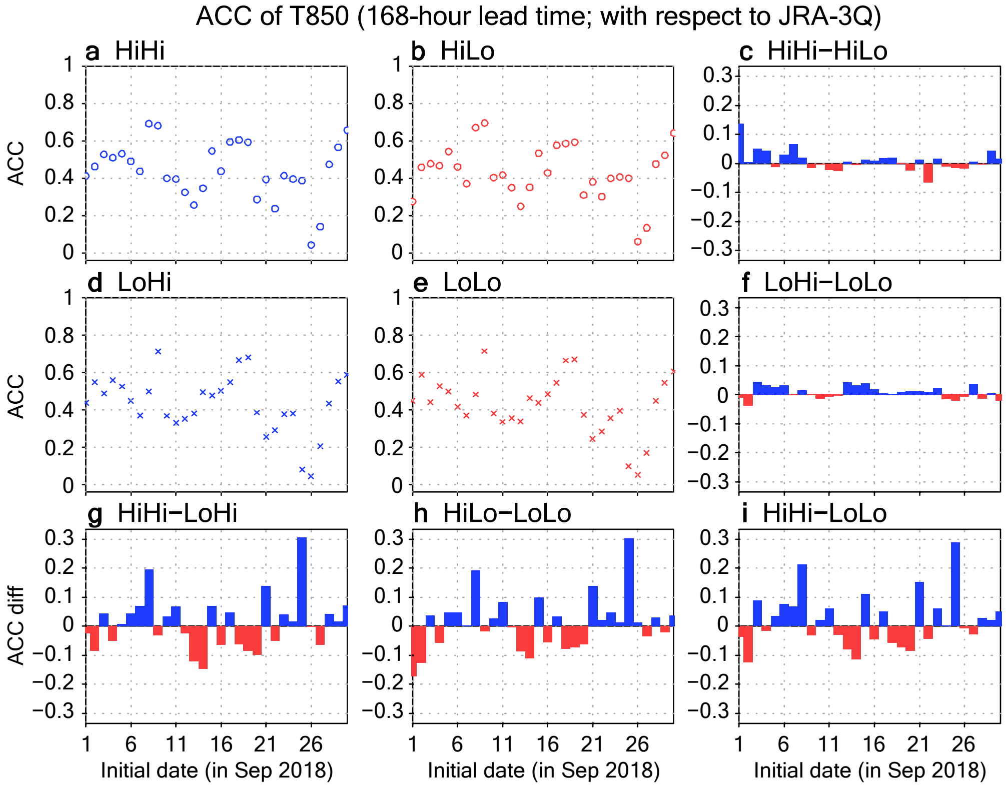

Figure S5. Same as Fig. S3 but for the anomaly correlation coefficient (ACC) scores of 850 hPa temperature (T850).

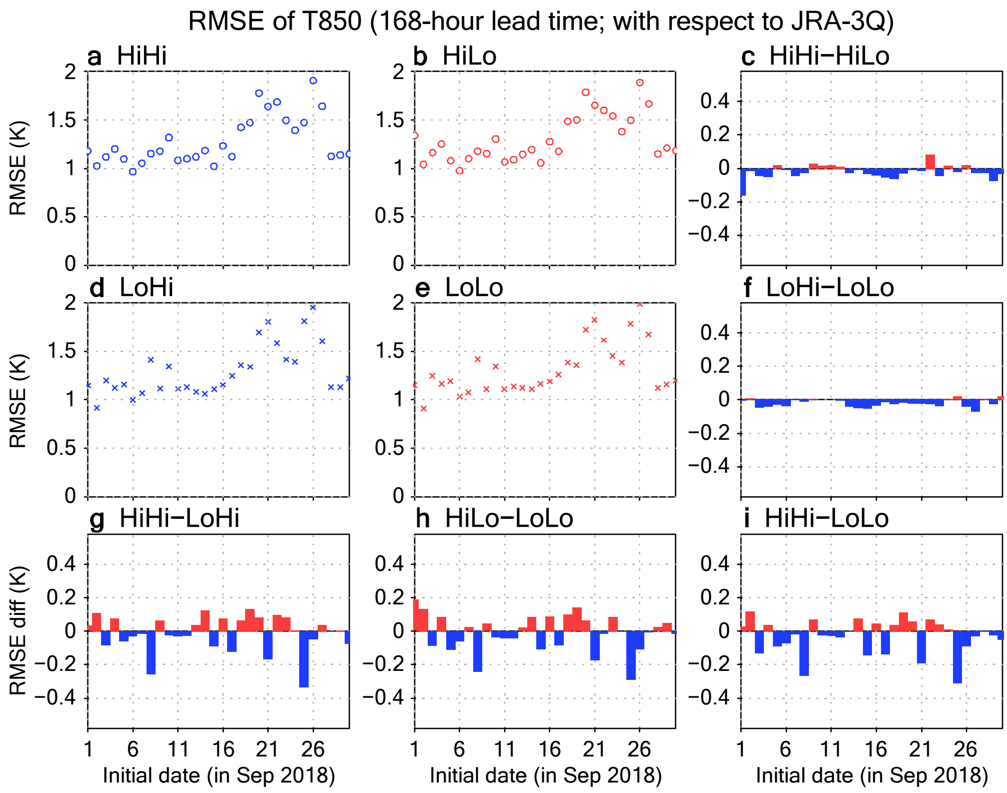

Figure S6. Same as Fig. S3 but for the root mean square error (RMSE) of 850 hPa temperature (T850).

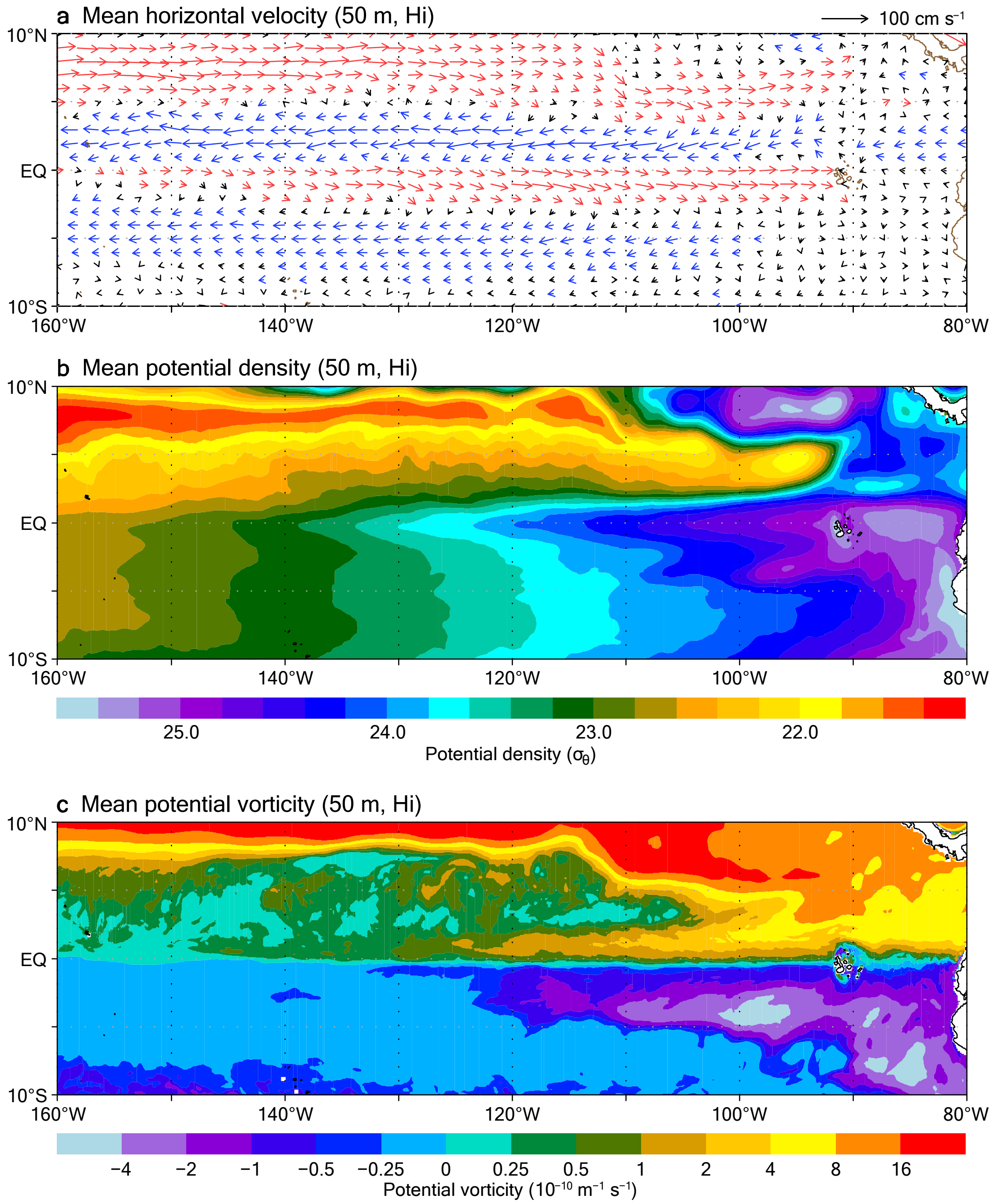

Figure S7. Background fields for calculating anomalous eddy fields (as used for the energy flux diagnostics), defined as the average of the initialization experiment results during August–October 2018. Examples are shown here for the high-resolution ocean initialization experiment (Hi) at 50 m depth. (**a**) Horizontal velocity. Relatively large zonal velocities (positive eastward) are indicated by blue ($\bar{u} < -10$ cm s^{-1}) and red ($\bar{u} >$

10 cm s^{-1}) arrows to highlight the equatorial zonal current system. (**b**) Potential density. (**c**) Potential vorticity, defined as $\frac{-1}{\rho_0}\frac{\partial\bar{\rho}}{\partial z}\left(f+\frac{\partial\bar{v}}{\partial x}-\frac{\partial\bar{u}}{\partial y}\right)$. Negative (positive) meridional gradients of potential vorticity are zonally distributed at about 2–3°N (farther north), as discussed in a previous study[25].
